# DNA Methylation Concurrence, Independent of DNA Methylation Ratios, Is Associated with Chromatin Accessibility and 3D Genome Architecture

**DOI:** 10.3390/ijms26157199

**Published:** 2025-07-25

**Authors:** Guian Zhang, Yixian Yang, Dan Cui, Jia Li

**Affiliations:** 1 State Key Laboratory of Respiratory Disease, Guangzhou Institute of Respiratory Health, Guangzhou Medical University, Guangzhou 510120, China; machfine@gmail.com (G.Z.);; 2 Guangzhou National Laboratory, Guangzhou 510005, China

**Keywords:** pan cancer, epigenomics, read-level DNA methylation pattern, scATAC-seq, histone modification, CTCF

## Abstract

Multiple metrics for read-level DNA methylation pattern analysis have provided new insights into DNA methylation modifications. However, the performance of these metrics and their relationship with DNA methylation ratios in identifying biologically meaningful regions have remained unclear. Here, we systematically benchmarked five read-level DNA methylation metrics using whole-genome bisulfite sequencing data from 59 individuals across six healthy tissue types and six tumor types. We found that DNA methylation concurrence (MCR) effectively captured tissue-specific features independent of the DNA methylation ratios. Regions that exhibited decreased MCR (MCDRs) in tumors were significantly enriched in promoter and intergenic regions and strongly overlapped with tumor-gained chromatin accessibility sites. The further analysis of histone modifications, including H3K4me3, H3K27ac, and H3K9ac, confirmed that MCDRs marked active gene regulatory elements. Motif enrichment analysis revealed a strong preference for CTCF binding within MCDRs. Additionally, 3D genome analysis supported a model in which MCDRs, independent of DNA methylation ratios, contribute to active gene regulation by facilitating CTCF binding and long-range chromatin interactions.

## 1. Introduction

DNA methylation is a fundamental epigenetic modification that regulates gene expression and preserves cellular identity, shaping lineage-specific transcriptional programs [1,2]. In normal cells, an active interplay between de novo methylation and demethylation contributes to the formation of cell-specific epigenetic signatures that guide differentiation [3]. However, in cancer, these finely tuned patterns become dysregulated, which results in widespread epigenetic reprogramming. Typically, such aberrations manifest as global hypomethylation of repetitive elements coupled with localized hypermethylation of gene promoters, a phenomenon that can silence tumor suppressors or activate oncogenes to drive tumor initiation and progression [4]. In lung, liver, kidney, gastric, colorectal, and breast cancers, extensive studies have demonstrated that methylation alterations are tightly associated with both the tumor behavior and clinical outcomes [5,6,7,8,9,10].

Despite the significant progress in understanding DNA methylation, most investigations still focus on the average methylation levels at individual CpG sites from bulk cell populations, potentially overlooking the inherent complexity of methylation patterns [11]. Each sequencing read from techniques such as whole-genome bisulfite sequencing (WGBS) captures the methylation status of CpG sites on a single chromosome within an individual cell, offering a detailed view at single-nucleotide resolution. This read-level methylation pattern has the potential to uncover subtle epigenetic variations that analyses based solely on average methylation levels might neglect, ultimately providing a more nuanced understanding of the role of DNA methylation in tumorigenesis and tumor progression.

Recent advances in read-level methylation analyses have given rise to multiple metrics that capture distinct facets of the epigenetic landscape. Notably, the methylation concurrence ratio (MCR) quantifies the co-occurrence of unmethylated CpGs within the partially methylated read, providing a sensitive measure to evaluate the dynamic interplay between active DNA methylation maintenance and demethylation processes. This approach has been reported to correlate more strongly with gene expression changes than average methylation levels, and can thus potentially reveals subtle epigenetic perturbations in cancer [12]. In addition, the methylation block score (MBS) and methylated haplotype load (MHL) are used to highlight contiguous regions of methylation and have already shown promise in noninvasive early cancer detection [13,14]. Meanwhile, entropy and the proportion of discordant reads (PDR) are increasingly recognized as measures of epigenetic heterogeneity, reflecting the degree of differences in the same methylation lever between regions [15].

Despite the growing adoption of these metrics, a significant gap remains in understanding how they integrate with chromatin accessibility profiles and 3D genome architecture. While single-cell ATAC-seq (scATAC-seq) provides a refined lens into chromatin state variation at the single-cell level [16], the interplay between these five DNA methylation patterns and local chromatin accessibility has not been systematically characterized, particularly in a pan-cancer setting. Moreover, how such methylation patterns may intersect with topologically associating domains (TADs), loop anchors, and other 3D structural features of the genome remains poorly defined. Dissecting these relationships is of critical importance, as higher-order chromatin conformations can bring distal regulatory elements into spatial proximity [17], potentially coordinating specific methylation patterns that govern gene expression in normal tissues and in tumorigenesis. Indeed, elucidating the connection between these read-level metrics, scATAC-derived chromatin accessibility, and 3D genome topology may offer unprecedented insights into the epigenetic regulation of cancer and has the potential to guide both mechanistic studies and clinical applications.

In this study, we systematically analyzed multi-omics data including WGBS, scATAC-seq, chromatin immunoprecipitation sequencing (ChIP-seq), and Hi-C across multiple cancer types and their matched normal epithelial tissues. We focused on five distinct read-level methylation patterns: MCR, MHL, MBS, entropy, and PDR. Our findings demonstrated that MCR serves as a robust metric for identifying tissue-specific methylation markers independently of the overall methylation levels. Methylation concurrence-depleted regions (MCDRs) in tumors were associated with active and enhancer-related histone modifications, as well as chromatin accessibility, suggesting that MCR reflects tumor-specific changes in methylation regulation. Moreover, we validated our findings in colorectal cancer (CRC) using single-cell whole-genome bisulfite sequencing (scWGBS), reinforcing the significance of MCR in tumor-specific epigenetic regulation. DNA motif analysis revealed significant enrichment of CTCF binding motifs in MCDRs, which was further validated by ChIP-seq and Hi-C data. This implied that MCR may reflect tumor progression through its role in modulating the 3D chromatin architecture. Overall, our findings not only deepened our understanding of how the methylation pattern drives tumorigenesis but also highlighted its potential as a novel diagnostic and prognostic biomarker for cancer.

## 2. Results

### 2.1. MCR Represents Tissue-Specific Epigenetic Features Independent of DNA Methylation Ratios

**Figure 1:**
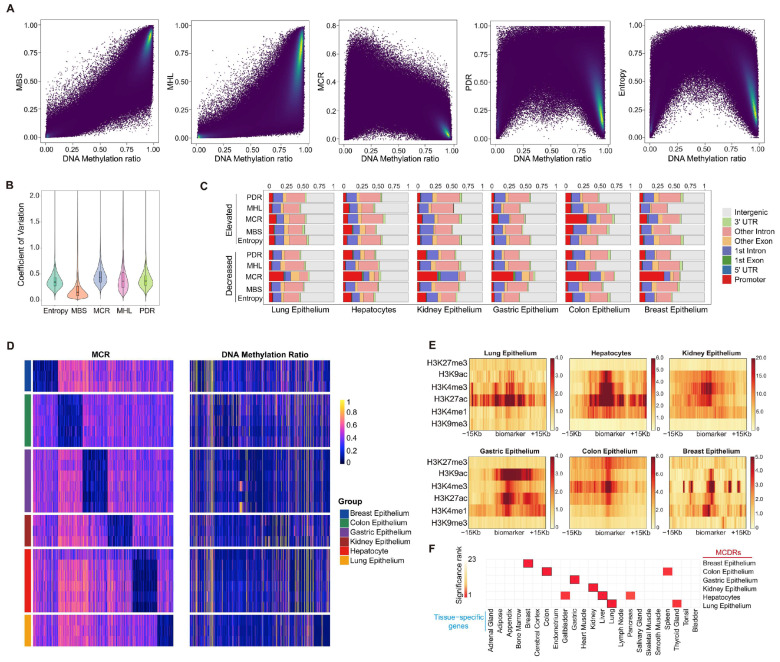
Characteristics of read-level methylation patterns and identification of MCDRs in six normal epithelial tissues. (**A**) Scatter plots showing the relationship between methylation level and five read-level metrics—MBS, MHL, MCR, PDR, and entropy in normal epithelial samples. (**B**) Coefficient of variation for each read-level metric across six purified normal epithelial tissues. (**C**) Bar charts illustrating the distribution of gene annotations corresponding to elevated or depleted methylation patterns. (**D**) Heatmap showing MCDRs with a specific decrease in MCR despite exhibiting no difference in methylation level. (**E**) Histone mark enrichment at MCDRs in six tissues. (**F**) Enrichment of MCDRs with tissue-specific genes.

Read-level analysis of DNA methylation data involves various algorithms. To evaluate their strengths and weaknesses, we performed a benchmark comparison of MBS, MHL, MCR, PDR, and entropy using epithelial cell DNA methylation data from normal tissues. We first segmented the human genome into regions with homogeneous DNA methylation ratios and then calculated five different read-level metrics for each region [18]. We observed that both MBS and MHL exhibited a positive correlation with DNA methylation ratios. In contrast, MCR, PDR, and entropy did not exhibit linear relationships with DNA methylation ratios, suggesting that these metrics may capture distinct aspects of DNA methylation information that are not directly correlated with the DNA methylation ratio (Figure 1A). Next, we examined how each of these read-level metrics varies among the six purified normal epithelial tissues. The coefficient of variation (CV) analysis revealed that MCR had the highest CV (Figure 1B), which emphasized its superior ability to differentiate between the various epithelial cell types when compared to the other metrics. Next, we identified regions exhibiting specific elevated or decreased tissue types in each of the five read-level metrics, independently of the DNA methylation ratios. Remarkably, about 30%–40% of the tissue-type specific MCDRs were strongly enriched in promoter regions, while other elevated or decreased metrics did not show similar distributions (Figure 1C). And those tissue-type specific MCDRs did not correlate with the DNA methylation ratios (Figure 1D). Additionally, these tissue-specific MCDRs were enriched for active histone marks such as H3K9ac, H3K4me3, H3K27ac, and H3K4me and depleted for repressive markers such as H3K9me3 and H3K27me3 (Figure 1E and Figure S1). Regions with other methylation patterns did not exhibit a consistent enrichment of histone marks (Figure S2).

For each of the six epithelial cell types, the gene set enrichment analysis using rGREAT demonstrated that regions exhibiting tissue-specific decreases in MCR were significantly enriched for genes specific to that cell type (Figure 1F and Figure S3). These findings suggested a potential role for MCR in the tissue-specific activation of gene regulation, uncovering a new aspect of DNA methylation regulation that is independent of DNA methylation ratios.

### 2.2. Disrupted DNA Methylation Concurrence in Tumors Identifies Regulatory Elements

**Figure 2:**
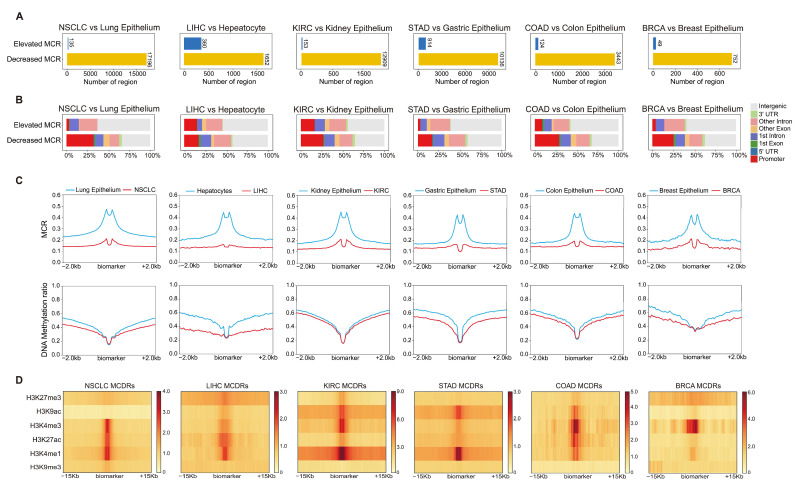
MCR depletion highlights regulatory regions in tumors with unchanged average methylation level. (**A**) The number of identified MCR regions that were not associated with the DNA methylation ratio. (**B**) The annotation of the regions identified by methylation in genomic features. (**C**) There was no detectable difference in the average DNA methylation level between tumor and normal tissues, but the MCR was significantly decreased. (**D**) Enrichment analysis of MCDRs for various histone modifications (H3K9ac, H3K4me3, H3K27ac, H3K4me1, H3K27me3, H3K9me3) in six tissue types.

To explore DNA methylation from a new perspective that is independent of the bulk DNA methylation ratios in tumors, we identified regions where the bulk DNA methylation ratio remined unchanged, yet the MCR was significantly altered in cancers. Notably, most of the regions showed decreased MCR in tumors, compared to the corresponding controls (Figure 2A). The annotation of regulatory elements revealed that approximately 20–30% of the MCDRs in tumors were strongly enriched at promoter regions, while 30–40% were enriched in intergenic regions (Figure 2B). Other metrics showed variable enrichment patterns (Figure S4A). The average DNA methylation ratios around these MCR-decreased regions in tumors were highly similar between the tumor and corresponding control (Figure 2C). We further investigated the histone marks enrichment on these MCR-decreased regions and found that they were significantly enriched for H3K9ac, H3K4me3, H3K27ac, and H3K4me1, which underscored their likely involvement in active transcription and enhancer function (Figure 2D). Similar to normal tissues, the MCR-increased regions did not show the consistent enrichment of histone marks (Figure S4B).

We compared the expression of Tss-proximal genes associated with MCDRs and found that these genes were significantly upregulated in tumors compared to normal tissues (Figure S5). In addition, we also analyzed the MCR changes in the traditionally defined hypermethylated and hypomethylated regions. The results showed that the MCR alteration and methylation levels in cancers were related but distinct phenomena (Figure S6). In sum, these findings demonstrated that MCDRs specifically mark regulatory regions in tumors where the average DNA methylation ratio remains unchanged, highlighting an additional layer of epigenetic complexity that the DNA methylation level alone may fail to capture.

### 2.3. Decreased DNA Methylation Concurrence Is Associated with the Acquisition of Open Chromatin in Tumors

**Figure 3:**
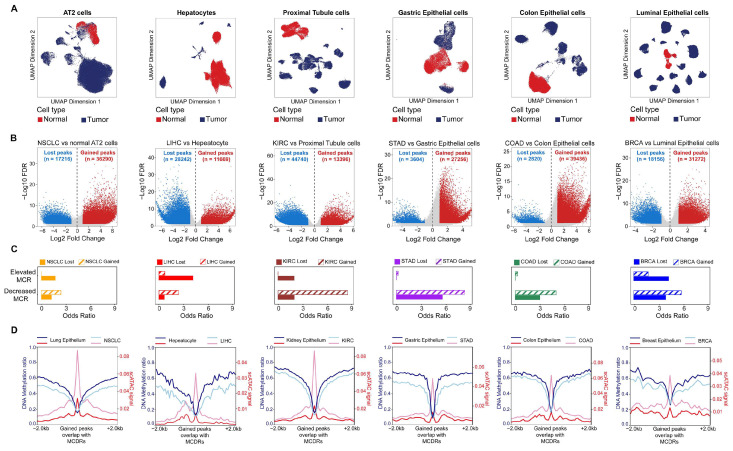
Single-cell ATAC-seq atlas of malignant and normal epithelium in six tissue types. (**A**) UMAP visualization of epithelial cells. (**B**) The identification of gained and lost peaks in malignant cells. (**C**) Enrichment of MCR regions with gained and lost peaks. (**D**) DNA methylation ratios and scATAC signals in the gained peaks overlapping MCDRs.

To further investigate the regulatory roles of these regions in gene expression, we collected scATAC data from malignant epithelial cells in six tissue types: lung adenocarcinoma (LUAD), hepatocellular carcinoma (LIHC), kidney renal clear cell carcinoma (KIRC), stomach adenocarcinoma (STAD), colon adenocarcinoma (COAD), and breast invasive carcinoma (BRCA), along with epithelial cells derived from corresponding normal tissues, which were used as controls. Given the diverse origins and datasets used in this study, each tissue type was separately annotated to ensure accurate cell-type classification (Figures S7 and S8). We then subset the epithelial cells and annotated them as normal or tumor groups (Figure 3A). We performed CNV analysis and retained only those epithelial cells with detectable copy number variations as malignant cells for subsequent analysis (Figure S9).

We next compared the chromatin accessibility profiles of malignant epithelial cells and normal epithelial cells through differential peak analysis. Using an absolute log2 (fold change) > 1 and FDR < 0.05 threshold, we identified two classes of differentially accessible regions: “gained peaks,” which are significantly more open in malignant cells, and “lost peaks,” which are significantly less open (Figure 3B). Next, we performed a LOLA enrichment analysis by intersecting tumor-specific elevated- or decreased-MCR regions with the scATAC-seq peaks mentioned above (Figure 3C). The results revealed that MCDRs within gained peaks attained the highest odds ratios, indicating that diminished DNA methylation concurrency preferred to be associated with increased chromatin accessibility in tumors. The DNA methylation ratios around these increased chromatin accessibility regions were highly similar between the normal and tumor cells and there was no change in the DNA methylation ratios on these gained peaks with MCDRs (Figure 3D). These data demonstrated that MCDRs in tumors gained chromatin accessibility in a manner that was independent of the DNA methylation ratios.

### 2.4. Dissecting MCR at the Single-Cell Level in Colorectal Cancer

**Figure 4:**
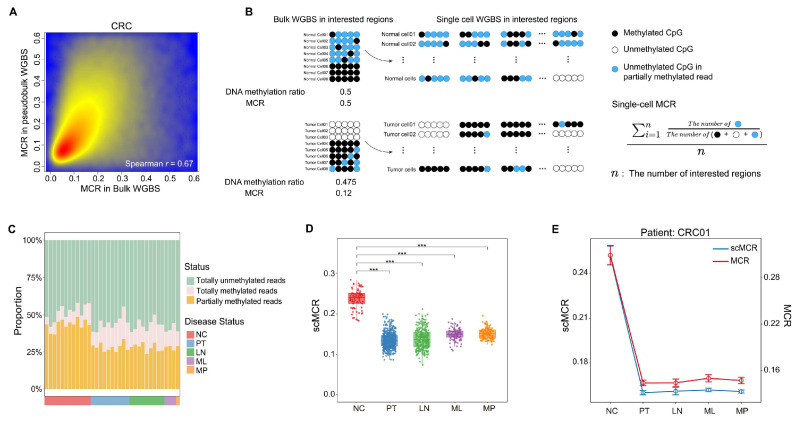
Single-cell WGBS validation of MCR-depleted regions in colorectal cancer. (**A**) The correlation between MCR of pseudobulk WBGS and MCR of real WGBS in CRC. (**B**) Schematic illustration of the computational algorithm of scMCR at the single-cell bisulfite-seq reads level. (**C**) The partial methylation frequencies of multiple CRC patients in MCDRs. (**D**) Box plot illustrating the scMCR of CRC within MCDRs (*** *p* < 0.001). (**E**) Longitudinal analysis of scMCR and MCR for patient CRC01.

To gain a deeper understanding of MCDRs at a single-cell resolution, we collected single-cell WGBS (scWGBS) data from CRC patients. This dataset included 87 adjacent normal epithelial cells (NCs), 545 primary tumor cells (PTs), 302 lymph node metastasis cells (LNs), 135 liver metastasis cells (MLs), and 113 tumor cells after liver metastasis treatment (MPs) [19]. We combined the scWGBS data into pseudobulk WGBS data, enabling the calculation of MCR. A scatterplot revealed a positive correlation between the values of MCR derived from the pseudobulk WGBS and those obtained from the real-bulk WBGS in CRC (Figure 4A). Considering that the reads used to calculate the MCR values were derived from each single cell, we adapted the MCR functions to single-cell MCR (scMCR). Specifically, we calculated the proportion of unmethylated CpGs in partially methylated reads mapped to colon cancer MCDRs by dividing the number of unmethylated CpGs by the total number of CpGs in each read mapped to these regions. This value was then averaged across all MCDR regions (Figure 4B). To characterize the DNA methylation status at the individual patient level, we stratified the data by patient and confirmed that the decline in partially methylated reads within the MCDRs was consistently observed across all CRC samples that were analyzed (Figure 4C). Then, we calculated the scMCR for each cell in each group. Consistent with the results obtained using bulk WGBS data, we observed a significant decrease in scMCR in the MCDRs for all CRC tumor cells, regardless of whether they originated from primary tumors, lymph node metastases, or liver metastases, when compared to normal colorectal epithelial cells. The results suggested that tumor cells at various stages exhibited a higher proportion of fully unmethylated reads and a lower proportion of fully methylated reads within the MCDRs, reflecting a reduced DNA methylation concurrence compared to normal cells (Figure 4D). A more detailed case study focused on patient CRC01, who provided samples spanning normal, primary tumor, lymph node metastasis, liver metastasis, and post-treatment stages (Figure 4E). Even as the disease progressed and treatments were administered, MCDRs in CRC01’s tumor cells persistently displayed a diminished fraction of partially methylated reads. These findings further support the idea that, at different stages of disease progression, tumor cells adopt a more uniform methylation pattern in the MCDR, exhibiting either fully unmethylated or mostly methylated CpGs. Quantifying the MCR values in CRC01’s tumor samples using pseudobulk WGBS similarly revealed a pronounced drop compared to normal tissue, which underlined the reproducibility and stability of the MCR decrease in these regions. Furthermore, we applied scWGBS data from prostate cancer (PCa) and obtained results similar to those obtained for CRC scWGBS, and we were also able to successfully validate these findings using the scMCR algorithm (Figure S10).

These analyses highlight the biological significance of MCDRs, demonstrating that the DNA methylation concurrence was consistently perturbed at a single-cell resolution during disease progression, with a strong correlation to MCR values derived from bulk WGBS data.

### 2.5. CTCF Binding at MCR-Decreased Regions Underlies Tumor-Specific Chromatin Reorganization and Gene Regulation

**Figure 5:**
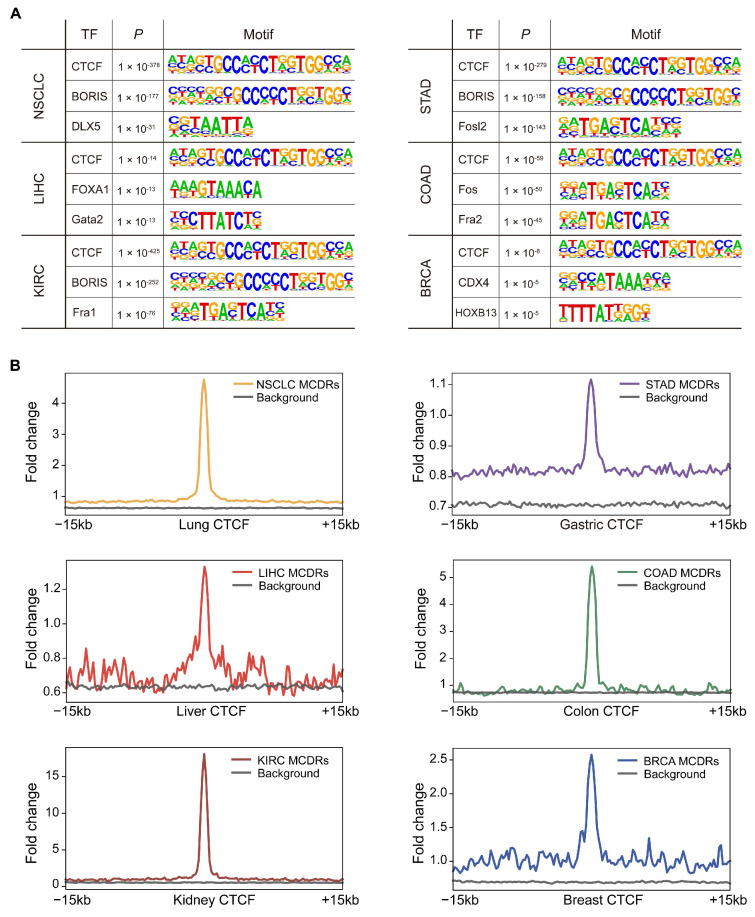
CTCF is the top-enriched motif at MCR-depleted regions in six tumor types. (**A**) HOMER motif enrichment analysis for MCDRs in six different cancers. (**B**) The enrichment of MCDRs with CTCF ChIP-seq peaks.

To further investigate the regulatory functions of MCDRs, we performed motif enrichment analysis using HOMER on MCDRs from six tumor types. Notably, CTCF emerged as the top-ranked motif in all six tumor types (Figure 5A), which highlighted its pivotal role for CTCF binding at sites where the average DNA methylation levels remain unchanged yet methylation concurrence was disrupted. Next, we analyzed CTCF binding signals in normal tissues around MCDRs and found that CTCF was significantly enriched at MCDRs compared to at random regions (Figure 5B).

**Figure 6:**
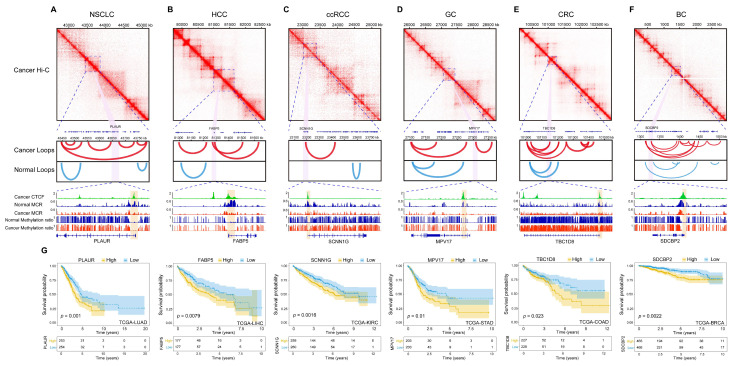
MCR-depleted loop anchors and CTCF binding drive 3D chromatin reorganization and clinical outcome. (**A**–**F**) Examples of six genes (one per tumor type) located within TADs and loop anchor regions. (**G**) Kaplan–Meier survival curves from TCGA data for these genes.

The results also suggested that CTCF was a well-known factor that was actively involved in shaping or maintaining the 3D chromatin architecture [20]. To gain deeper insight into how these MCDRs are involved in higher-order chromatin organization, we exemplified one gene in each tumor type (Figure 6A–F). Each gene resided within a defined TAD and was situated at loop anchor regions, as evidenced by Hi-C data. Notably, the number of loops involving these genes was increased in the tumor state compared with the number in normal tissues, which suggested enhanced long-range contacts that could facilitate dysregulated gene expression. The promoters of these genes were enriched in CTCF peaks, exhibiting decreased MCR in tumors, and showed minimal changes in their DNA methylation ratios. This supports the notion that CTCF-bound loop anchors, which are independent of DNA methylation ratios but associated with decreased MCR, might play a central role in reorganizing the local chromatin architecture. By bringing distal regulatory elements into closer spatial proximity, these loops could either activate or repress key oncogenic or tumor-suppressive pathways, thereby shaping cancer phenotypes. We next evaluated the clinical impact of these genes by performing TCGA survival analyses. As shown in Figure 6G, these genes were significantly correlated with patient outcomes, which implied that their dysregulation at MCR-depleted CTCF-bound loop anchors may contribute to tumor progression or aggressiveness. Additionally, online analyses using UALCAN confirmed that these genes are differentially expressed between tumor and normal tissues (Figure S11), which reinforces their potential relevance as molecular biomarkers or therapeutic targets [21].

Overall, our findings highlight that regions with decreased MCR, independently of the DNA methylation ratios, frequently converge on CTCF-binding sites at loop anchors and TAD boundaries. This may contribute to a permissive or altered chromatin environment in cancer cells, shaping tumor-specific gene regulatory programs with clinically significant consequences.

## 3. Discussion

In this study, we performed a comprehensive pan-cancer analysis that integrated WGBS and scATAC-seq data from six different tumor types and their corresponding normal epithelia. Previously, DNA methylation-related regulatory elements were mainly identified by the DNA methylation ratios [22]. Our work focused on five distinct read-level methylation metrics, with special emphasis on MCR. By examining regions where the average DNA methylation ratios remained unchanged yet the MCR was significantly altered, we uncovered MCDRs that recurrently overlap with promoter or enhancer elements and harbor active histone marks. Furthermore, these regions exhibited tissue-specific histone modifications and were enriched for genes with tissue-specific expression, which underscored their specialized regulatory roles. In tumor specimens, the MCDRs predominantly resided within accessible chromatin regions. Single-cell analyses in CRC further validated these findings, demonstrating that a reduction in MCR was a consistent marker of tumor progression. Notably, these regions also exhibited strong enrichment in CTCF-binding sites and coincided with changes in the 3D chromatin architecture, such as changes in TAD boundaries and loop anchors. Our findings highlighted MCR depletion as a unique epigenetic signature that was not captured by average methylation ratios alone. In particular, the pronounced changes observed at MCDRs suggest that methylation concurrency might play a direct role in shaping tissue-specific regulatory networks as cells transition from normal epithelium to malignant states. Additionally, the overlap of MCDRs with CTCF-bound loop anchors strongly suggested that MCR depletion was tied to 3D genome reorganization, whereby emerging or altered loops can bring distal regulatory elements into proximity to activate or repress key oncogenic or tumor-suppressive pathways. This insight was further bolstered by clinical relevance, as key genes located within MCDRs correlate with survival outcomes and exhibit differential expression in large patient cohorts. Thus, MCR held promise for refining current models of tumor evolution and for identifying potential biomarkers of cancer risk or therapeutic response.

We focused on six common epithelial-origin cancers, each of which was systematically paired with its established cellular origins for WGBS profiling. Our tissue-specific sampling strategy included alveolar epithelial cells for lung adenocarcinoma [23,24], hepatocytes for hepatocellular carcinoma [25], proximal tubule epithelial cells for kidney renal clear cell carcinoma [26,27], gastric and colon epithelial cells for gastric adenocarcinoma and colorectal cancer [28,29], and luminal epithelial cells for breast cancer [30,31]. The initial analysis demonstrated that MCDRs, identified independently of the average DNA methylation ratio, exhibited remarkable tissue specificity. These MCDRs, in normal tissues, showed significant enrichment for chromatin activation marks (H3K27ac, H3K9ac, and H3K4me3) specific to their tissue of origin. We further validated this finding using scATAC-seq data from matching epithelial cell populations, which showed robust tissue-specific accessibility signals at these MCDRs. This multi-layered approach confirmed that MCDRs could capture essential cell-of-origin characteristics and underscored the importance of accurate tissue-matched comparisons in epigenetic studies. Having established the tissue-specific nature of MCR, we next explored the mechanistic underpinnings of MCR and how it intersected with the chromatin accessibility and 3D chromatin architecture to drive tumor-specific regulatory programs.

A central theme emerging from our study was how MCR depletion integrated with 3D organization of the genome to facilitate tumor-specific regulatory programs. Our data revealed that MCDRs frequently overlapped with CTCF-binding sites, TAD boundaries, and loop anchors, which are key structural elements that are known to partition the genome into regulatory neighborhoods. In normal cells, these architectural features helped maintain proper gene expression by isolating enhancers and promoters within defined spatial compartments [32]. However, when the MCR decreases within these domains, the epigenetic landscape might become more polarized, tending toward fully methylated or fully unmethylated CpGs and potentially altering the formation or stability of chromatin loops. Indeed, the gained peaks identified in the tumor scATAC-seq data often coincided with MCDRs, which supported the idea that changes in local methylation concurrency could loosen or remodel nucleosome packing, thereby granting greater accessibility to transcription factors. By extension, CTCF, a master architectural protein [33], appeared to be pivotal for tethering loop anchors in these MCR-decreased regions. The heightened presence of CTCF motifs within MCDRs suggests that MCR depletion might facilitate or accompany long-range interactions, allowing enhancers and promoters to establish novel contacts. This phenomenon could drive both the activation of oncogenes and the repression of tumor-suppressor genes, depending on the 3D chromatin architecture and the specific regulatory modules brought into proximity [34].

Our observation that the loops increased in number or span in tumors implies the dynamic reorganization of chromatin domains, which is possibly driven by the epigenetic instability that accompanies disease progression. These newly formed or strengthened loops might further reinforce dysregulated transcriptional programs, which could culminate in phenotypic diversity and enhanced metastatic potential [35,36]. Additionally, the consistent depletion of MCR across multiple tumor stages, which was confirmed through scWGBS in CRC, indicated that epigenetic heterogeneity within TADs and loop anchors is not merely a late-stage event but might emerge early and persist throughout the disease’s evolution. Taken together, these findings positioned MCDRs as critical hotspots where epigenetic and 3D structural alterations converge to shape the tumor epigenome in a tissue-specific manner. In essence, DNA methylation was not solely a linear “on/off” mechanism [37]. Rather, the co-occurrence of CpGs in methylated or unmethylated states profoundly influenced chromatin folding and the usage of regulatory elements. Understanding the interplay between MCR depletion, CTCF-mediated looping, and histone modifications provided new avenues for dissecting how epigenetic perturbations orchestrated oncogenic pathways. This mechanistic framework might guide future studies that aim to exploit 3D genome integrity as part of targeted epigenetic therapies [38].

In summary, this study integrates multi-omics and single-cell approaches to identify MCDRs as a tissue-specific epigenetic signature that is distinct from DNA methylation ratios. We further revealed that MCR depletion uncovered pivotal regulatory elements linked to chromatin accessibility and the 3D genome architecture. By demonstrating clinical relevance in multiple cancers, MCR was expected to be a powerful read-level epigenetic metric with potential biomarker and therapeutic utility.

## 4. Materials and Methods

### 4.1. Data Sources

All data used in this study are publicly available. We collected a total of 59 WGBS samples from six tissue types, comprising 26 purified normal epithelial samples: alveolar epithelial cells (*n* = 3), hepatocytes (*n* = 6), kidney tubule epithelial cells (*n* = 3), gastric epithelial cells (*n* = 6), colonic epithelial cells (*n* = 5), and luminal epithelial cells (*n* = 3), as well as 33 corresponding cancer samples, including NSCLC (*n* = 6), LIHC (*n* = 6), KIRC (*n* = 3), STAD (*n* = 3), COAD (*n* = 10), and BRCA (*n* = 5). In addition, we gathered 87 adjacent normal colonic epithelial cells and 1095 scWGBS profiles that span various disease stages in colorectal cancer (Tables S1 and S2). For chromatin accessibility analyses, we compiled 109 scATAC-seq datasets, with normal tissue samples from lung (*n* = 11), liver (*n* = 9), kidney (*n* = 4), stomach (*n* = 4), colon (*n* = 7), and breast (*n* = 5), and tumor samples from LUAD (*n* = 11), LIHC (*n* = 13), KIRC (*n* = 22), STAD (*n* = 2), COAD (*n* = 5), and BRCA (*n* = 16) (Table S3). ChIP-seq data for histone modifications and CTCF were obtained from ENCODE, GEO, and Roadmap [39] (Table S4). The Hi-C datasets used in this study are listed in Table S5. RNA-seq data and accompanying clinicopathological information for the six cancer types were obtained from The Cancer Genome Atlas (TCGA) database (Table S5).

### 4.2. DNA Methylation Data Analysis

Downloaded FASTQ files were first subjected to quality control using FastQC (v0.11.8). Sequencing adapters and low-quality bases were trimmed using TrimGalore (v0.6.4). Trimmed sequences were aligned to the human reference genome (hg19) using BSMAP with default parameters [40]. Duplicate reads were identified and removed using the MarkDuplicates function from the Picard toolkit (v2.27.4), with the parameter REMOVE_DUPLICATES = true. DNA methylation ratios at each CpG site were extracted using MethyDackel (v0.6.1) with default parameters. BAM files were converted to MHAP format using mHapSuite (v2.1) for downstream analyses. For WGBS data available only in PAT format, files were similarly converted to MHAP format using mHapSuite with parameters ‘--pat’ to facilitate subsequent analyses.

### 4.3. Identification of Regions Associated with DNA Methylation Patterns Independent of DNA Methylation Ratios

We first segmented the entire genome into homogeneously methylated blocks using the segmentation feature of wgbstools with parameters ‘—min_cpg 4’ [41], retaining only those blocks that covered at least four CpG sites. Next, we calculated the methylation patterns for these blocks, including entropy, MBS, MCR, MHL, and PDR, using the stat function in mHapSuite, retaining only values derived from regions with at least 10 reads. Regions showing significant differences in DNA methylation ratios (*p* < 0.05, Wilcoxon rank-sum test) were excluded, while regions exhibiting significant differences only in methylation patterns (*p* < 0.05, Wilcoxon rank-sum test) were retained. Furthermore, regions with differences in DNA methylation ratios less than 0.2 and differences in methylation patterns greater than 0.2 between groups were selected for subsequent analysis.

### 4.4. Single-Cell ATAC Data Analysis

We conducted scATAC-seq data analysis using ArchR(v1.0.2) [42]. First, we loaded the hg38 reference genome by invoking addArchRGenome and filtered the data for high quality cells by retaining those with a TSS enrichment score ≥ 4 and at least 3000 unique fragments per cell. Next, dimensionality reduction was performed using addIterativeLSI (4 iterations, 15,000 variable features, using the first 20 LSI dimensions), followed by visualization and manual cell annotation based on gene activity scores and marker gene expression, with a focus on identifying epithelial cell populations. For the epithelial cell population, we conducted CNV analysis to identify malignant cells: blacklist filtered fragments were counted in 10 Mb genomic bins, bins with GC content < 30% were removed, and GC correction was performed using modal regression. Cells with more than 5% missing data after correction were excluded. Subsequently, the smooth CNA function (smooth.region = 4) from the DNACopy (v1.76) R package was used to smooth the corrected counts, which were then mean-normalized per cell, and this was followed by clustering using Seurat and segmentation with DNACopy’s segment function (alpha = 0.2, nperm = 10) and mergeLevels function [43]. Finally, peak calling was performed with MACS2 [44] to define differential peaks as those with an absolute log2 (fold change) > 1 and FDR < 0.05; peaks were mapped from hg38 to hg19 using UCSC liftOver [45]. The reads in normalized bigwig files were generated using ArchR’s getGroupBW function with a tile size of 100 bp and a maximum of 2000 cells per cluster to support subsequent data visualization.

### 4.5. Motif Analysis

We employed the HOMER [46] function “findMotifsGenome.pl” with the “-size given” parameter to identify known motifs within the MCDRs. For each cancer, the top three most significant motifs (*p* < 0.05) were selected for presentation.

### 4.6. Enrichment Analysis of Tissue-Specific Genes

As demonstrated in previous studies [47], we identified tissue-specific gene sets for each tissue type using the Human Protein Atlas dataset available in the TissueEnrich (v1.22) R package [48]. Subsequently, we assessed the enrichment of MCDRs in these tissue-specific gene sets using the default parameters in rGREAT [49]. The tissue-specific enrichment of MCDRs was systematically ranked across 23 distinct tissue types based on statistical significance.

### 4.7. Enrichment Analysis for Gained and Lost Peak Regions

LOLA (locus overlap analysis, v1.32) is an R package designed for enrichment analysis of genomic regions [50]. We utilized LOLA to evaluate the enrichment of identified MCDRs within gained and lost peaks, using all regions without significant differences in DNA methylation ratios as the background set. Enrichment analyses were performed using the default parameters, and statistical significance was assessed via a default one-sided Fisher’s exact test.

### 4.8. ChIP-Seq Data Analysis

As described above, downloaded ChIP-seq FASTQ files underwent trimming of adapters and low-quality reads using TrimGalore. Alignment to the human genome (hg19) was carried out using Bowtie2 with default parameters [51], which was followed by the removal of duplicate sequences using Picard. MACS2 (version 2.2.7.1) was then employed to identify peaks [44], with a false discovery rate threshold of FDR < 0.05 being applied. We utilized our in-house Mmint “https://github.com/lijiacd985/Mmint” (accessed on 27 July 2024) “horizonalHeatmap” function to simultaneously visualize the enrichment of multiple histone modifications in a heatmap. To depict CTCF signals, we employed the “multibw2Bed” function within Mmint. Additionally, random regions were generated as background controls using the shuffle function in BEDTools [52].

### 4.9. Hi-C Data Analysis

We downloaded all Hi-C data used in this study in the hic file format from ENCODE (hg38) and GEO (hg19). To match the hic files from ENCODE, we used UCSC liftOver to convert MCDRs from hg19 to hg38. For datasets that provided corresponding loop files in bedpe format, these were directly utilized for downstream analyses. For datasets without such files, we detected loops using the hicDetectLoops function in the HiCExplorer software (v3.7.5) [53]. We selected MCDRs located within chromatin loop anchors, identifying regions with potential long-range physical contact with genes. Then, we specifically pinpointed those situated at or near the promoters of known genes. We analyzed the expression and prognostic value of genes using data from TCGA. Visualization was performed using the Juicebox tool [54].

## Data Availability

All data reported in this article will be shared upon request.

## Supplementary Materials

The following supporting information can be downloaded at: https://www.mdpi.com/article/10.3390/ijms26157199/s1.

## References

[1] Razin A., Riggs A.D. (1980). DNA Methylation and Gene Function. Science.

[2] Schübeler D. (2015). Function and Information Content of DNA Methylation. Nature.

[3] Moore L.D., Le T., Fan G. (2013). DNA Methylation and Its Basic Function. Neuropsychopharmacology.

[4] Shen H., Laird P.W. (2013). Interplay between the Cancer Genome and Epigenome. Cell.

[5] Rauch T.A., Wang Z., Wu X., Kernstine K.H., Riggs A.D., Pfeifer G.P. (2012). DNA Methylation Biomarkers for Lung Cancer. Tumor Biol..

[6] Zhu J.D. (2005). The Altered DNA Methylation Pattern and Its Implications in Liver Cancer. Cell Res..

[7] Shenoy N., Vallumsetla N., Zou Y., Galeas J.N., Shrivastava M., Hu C., Susztak K., Verma A. (2015). Role of DNA Methylation in Renal Cell Carcinoma. J. Hematol. Oncol..

[8] Watanabe Y., Kim H.S., Castoro R.J., Chung W., Estecio M.R.H., Kondo K., Guo Y., Ahmed S.S., Toyota M., Itoh F. (2009). Sensitive and Specific Detection of Early Gastric Cancer with DNA Methylation Analysis of Gastric Washes. Gastroenterology.

[9] Yagi K., Akagi K., Hayashi H., Nagae G., Tsuji S., Isagawa T., Midorikawa Y., Nishimura Y., Sakamoto H., Seto Y. (2010). Three DNA Methylation Epigenotypes in Human Colorectal Cancer. Clin. Cancer Res..

[10] Liu J., Zhao H., Huang Y., Xu S., Zhou Y., Zhang W., Li J., Ming Y., Wang X., Zhao S. (2021). Genome-Wide Cell-Free DNA Methylation Analyses Improve Accuracy of Non-Invasive Diagnostic Imaging for Early-Stage Breast Cancer. Mol. Cancer.

[11] Hong Y., Liu L., Feng Y., Zhang Z., Hou R., Xu Q., Shi J. (2023). mHapBrowser: A Comprehensive Database for Visualization and Analysis of DNA Methylation Haplotypes. Nucleic Acids Res..

[12] Shi J., Xu J., Chen Y.E., Li J.S., Cui Y., Shen L., Li J.J., Li W. (2021). The Concurrence of DNA Methylation and Demethylation Is Associated with Transcription Regulation. Nat. Commun..

[13] Liang N., Li B., Jia Z., Wang C., Wu P., Zheng T., Wang Y., Qiu F., Wu Y., Su J. (2021). Ultrasensitive Detection of Circulating Tumour DNA via Deep Methylation Sequencing Aided by Machine Learning. Nat. Biomed. Eng..

[14] Guo S., Diep D., Plongthongkum N., Fung H.-L., Zhang K., Zhang K. (2017). Identification of Methylation Haplotype Blocks Aids in Deconvolution of Heterogeneous Tissue Samples and Tumor Tissue-of-Origin Mapping from Plasma DNA. Nat. Genet..

[15] Landau D.A., Clement K., Ziller M.J., Boyle P., Fan J., Gu H., Stevenson K., Sougnez C., Wang L., Li S. (2014). Locally Disordered Methylation Forms the Basis of Intra-Tumor Methylome Variation in Chronic Lymphocytic Leukemia. Cancer Cell.

[16] Sinha S., Satpathy A.T., Zhou W., Ji H., Stratton J.A., Jaffer A., Bahlis N., Morrissy S., Biernaskie J.A. (2021). Profiling Chromatin Accessibility at Single-Cell Resolution. Genom. Proteom. Bioinform..

[17] Yao L., Berman B.P., Farnham P.J. (2015). Demystifying the Secret Mission of Enhancers: Linking Distal Regulatory Elements to Target Genes. Crit. Rev. Biochem. Mol. Biol..

[18] Loyfer N., Magenheim J., Peretz A., Cann G., Bredno J., Klochendler A., Fox-Fisher I., Shabi-Porat S., Hecht M., Pelet T. (2023). A DNA Methylation Atlas of Normal Human Cell Types. Nature.

[19] Bian S., Hou Y., Zhou X., Li X., Yong J., Wang Y., Wang W., Yan J., Hu B., Guo H. (2018). Single-Cell Multiomics Sequencing and Analyses of Human Colorectal Cancer. Science.

[20] Andreu M.J., Alvarez-Franco A., Portela M., Gimenez-Llorente D., Cuadrado A., Badia-Careaga C., Tiana M., Losada A., Manzanares M. (2022). Establishment of 3D Chromatin Structure after Fertilization and the Metabolic Switch at the Morula-to-Blastocyst Transition Require CTCF. Cell Rep..

[21] Chandrashekar D.S., Karthikeyan S.K., Korla P.K., Patel H., Shovon A.R., Athar M., Netto G.J., Qin Z.S., Kumar S., Manne U. (2022). UALCAN: An Update to the Integrated Cancer Data Analysis Platform. Neoplasia.

[22] Guo H., Vuille J.A., Wittner B.S., Lachtara E.M., Hou Y., Lin M., Zhao T., Raman A.T., Russell H.C., Reeves B.A. (2023). DNA Hypomethylation Silences Anti-Tumor Immune Genes in Early Prostate Cancer and CTCs. Cell.

[23] Zacharias W.J., Frank D.B., Zepp J.A., Morley M.P., Alkhaleel F., Kong J., Zhou S., Cantu E., Morrisey E.E. (2018). Regeneration of the Lung Alveolus by an Evolutionarily Conserved Epithelial Progenitor. Nature.

[24] Treutlein B., Brownfield D.G., Wu A.R., Neff N.F., Mantalas G.L., Espinoza F.H., Desai T.J., Krasnow M.A., Quake S.R. (2014). Reconstructing Lineage Hierarchies of the Distal Lung Epithelium Using Single-Cell RNA-Seq. Nature.

[25] Mu X., Español-Suñer R., Mederacke I., Affò S., Manco R., Sempoux C., Lemaigre F.P., Adili A., Yuan D., Weber A. (2015). Hepatocellular Carcinoma Originates from Hepatocytes and Not from the Progenitor/Biliary Compartment. J. Clin. Investig..

[26] Hsieh J.J., Purdue M.P., Signoretti S., Swanton C., Albiges L., Schmidinger M., Heng D.Y., Larkin J., Ficarra V. (2017). Renal Cell Carcinoma. Nat. Rev. Dis. Primer.

[27] Young M.D., Mitchell T.J., Vieira Braga F.A., Tran M.G.B., Stewart B.J., Ferdinand J.R., Collord G., Botting R.A., Popescu D.-M., Loudon K.W. (2018). Single-Cell Transcriptomes from Human Kidneys Reveal the Cellular Identity of Renal Tumors. Science.

[28] Tan P., Yeoh K.-G. (2015). Genetics and Molecular Pathogenesis of Gastric Adenocarcinoma. Gastroenterology.

[29] Joanito I., Wirapati P., Zhao N., Nawaz Z., Yeo G., Lee F., Eng C.L.P., Macalinao D.C., Kahraman M., Srinivasan H. (2022). Single-Cell and Bulk Transcriptome Sequencing Identifies Two Epithelial Tumor Cell States and Refines the Consensus Molecular Classification of Colorectal Cancer. Nat. Genet..

[30] Molyneux G., Geyer F.C., Magnay F.-A., McCarthy A., Kendrick H., Natrajan R., MacKay A., Grigoriadis A., Tutt A., Ashworth A. (2010). BRCA1 Basal-like Breast Cancers Originate from Luminal Epithelial Progenitors and Not from Basal Stem Cells. Cell Stem Cell.

[31] Sayaman R.W., Miyano M., Carlson E.G., Senapati P., Zirbes A., Shalabi S.F., Todhunter M.E., Seewaldt V.E., Neuhausen S.L., Stampfer M.R. (2024). Luminal Epithelial Cells Integrate Variable Responses to Aging into Stereotypical Changes that Underlie Breast Cancer Susceptibility. eLife.

[32] Seitan V.C., Faure A.J., Zhan Y., McCord R.P., Lajoie B.R., Ing-Simmons E., Lenhard B., Giorgetti L., Heard E., Fisher A.G. (2013). Cohesin-Based Chromatin Interactions Enable Regulated Gene Expression within Preexisting Architectural Compartments. Genome Res..

[33] Kitamura Y., Takahashi K., Maezawa S., Munakata Y., Sakashita A., Katz S.P., Kaplan N., Namekawa S.H. (2025). CTCF-Mediated 3D Chromatin Sets up the Gene Expression Program in the Male Germline. Nat. Struct. Mol. Biol..

[34] Deng S., Feng Y., Pauklin S. (2022). 3D Chromatin Architecture and Transcription Regulation in Cancer. J. Hematol. Oncol..

[35] Wang M., Sunkel B.D., Ray W.C., Stanton B.Z. (2022). Chromatin Structure in Cancer. BMC Mol. Cell Biol..

[36] Monteagudo-Sánchez A., Noordermeer D., Greenberg M.V.C. (2024). The Impact of DNA Methylation on CTCF-Mediated 3D Genome Organization. Nat. Struct. Mol. Biol..

[37] Johnson N.D., Wiener H.W., Smith A.K., Nishitani S., Absher D.M., Arnett D.K., Aslibekyan S., Conneely K.N. (2017). Non-Linear Patterns in Age-Related DNA Methylation May Reflect CD4+ T Cell Differentiation. Epigenetics.

[38] Achinger-Kawecka J., Stirzaker C., Portman N., Campbell E., Chia K.-M., Du Q., Laven-Law G., Nair S.S., Yong A., Wilkinson A. (2024). The Potential of Epigenetic Therapy to Target the 3D Epigenome in Endocrine-Resistant Breast Cancer. Nat. Struct. Mol. Biol..

[39] Kundaje A., Meuleman W., Ernst J., Bilenky M., Yen A., Heravi-Moussavi A., Kheradpour P., Zhang Z., Wang J., Roadmap Epigenomics Consortium (2015). Integrative Analysis of 111 Reference Human Epigenomes. Nature.

[40] Xi Y., Li W. (2009). BSMAP: Whole Genome Bisulfite Sequence MAPping Program. BMC Bioinform..

[41] Loyfer N., Rosenski J., Kaplan T. (2024). Wgbstools: A Computational Suite for DNA Methylation Sequencing Data Representation, Visualization, and Analysis. bioRxiv.

[42] Granja J.M., Corces M.R., Pierce S.E., Bagdatli S.T., Choudhry H., Chang H.Y., Greenleaf W.J. (2021). ArchR Is a Scalable Software Package for Integrative Single-Cell Chromatin Accessibility Analysis. Nat. Genet..

[43] Sundaram L., Kumar A., Zatzman M., Salcedo A., Ravindra N., Shams S., Louie B.H., Bagdatli S.T., Myers M.A., Sarmashghi S. (2024). Single-Cell Chromatin Accessibility Reveals Malignant Regulatory Programs in Primary Human Cancers. Science.

[44] Zhang Y., Liu T., Meyer C.A., Eeckhoute J., Johnson D.S., Bernstein B.E., Nusbaum C., Myers R.M., Brown M., Li W. (2008). Model-Based Analysis of ChIP-Seq (MACS). Genome Biol..

[45] Kent W.J., Sugnet C.W., Furey T.S., Roskin K.M., Pringle T.H., Zahler A.M., Haussler D. (2002). The Human Genome Browser at UCSC. Genome Res..

[46] Heinz S., Benner C., Spann N., Bertolino E., Lin Y.C., Laslo P., Cheng J.X., Murre C., Singh H., Glass C.K. (2010). Simple Combinations of Lineage-Determining Transcription Factors Prime Cis-Regulatory Elements Required for Macrophage and B Cell Identities. Mol. Cell.

[47] Feng Y., Zhang Z., Hong Y., Ding Y., Liu L., Gao S., Fang H., Shi J. (2023). A DNA Methylation Haplotype Block Landscape in Human Tissues and Preimplantation Embryos Reveals Regulatory Elements Defined by Comethylation Patterns. Genome Res..

[48] Jain A., Tuteja G. (2019). TissueEnrich: Tissue-Specific Gene Enrichment Analysis. Bioinforma. Oxf. Engl..

[49] McLean C.Y., Bristor D., Hiller M., Clarke S.L., Schaar B.T., Lowe C.B., Wenger A.M., Bejerano G. (2010). GREAT Improves Functional Interpretation of Cis-Regulatory Regions. Nat. Biotechnol..

[50] Sheffield N.C., Bock C. (2016). LOLA: Enrichment Analysis for Genomic Region Sets and Regulatory Elements in R and Bioconductor. Bioinformatics.

[51] Langmead B., Salzberg S.L. (2012). Fast Gapped-Read Alignment with Bowtie 2. Nat. Methods.

[52] Quinlan A.R., Hall I.M. (2010). BEDTools: A Flexible Suite of Utilities for Comparing Genomic Features. Bioinforma. Oxf. Engl..

[53] Wolff J., Backofen R., Grüning B. (2022). Loop Detection Using Hi-C Data with HiCExplorer. GigaScience.

[54] Durand N.C., Shamim M.S., Machol I., Rao S.S.P., Huntley M.H., Lander E.S., Aiden E.L. (2016). Juicer Provides a One-Click System for Analyzing Loop-Resolution Hi-C Experiments. Cell Syst..

