# Supplementary figures and images for: DNA Methylation Concurrence, Independent of DNA Methylation Ratios, Is Associated with Chromatin Accessibility and 3D Genome Architecture
Source: Int J Mol Sci. 2025 Jul 25;26(15):7199. doi: 10.3390/ijms26157199 (PMC12346627; doi:10.3390/ijms26157199)

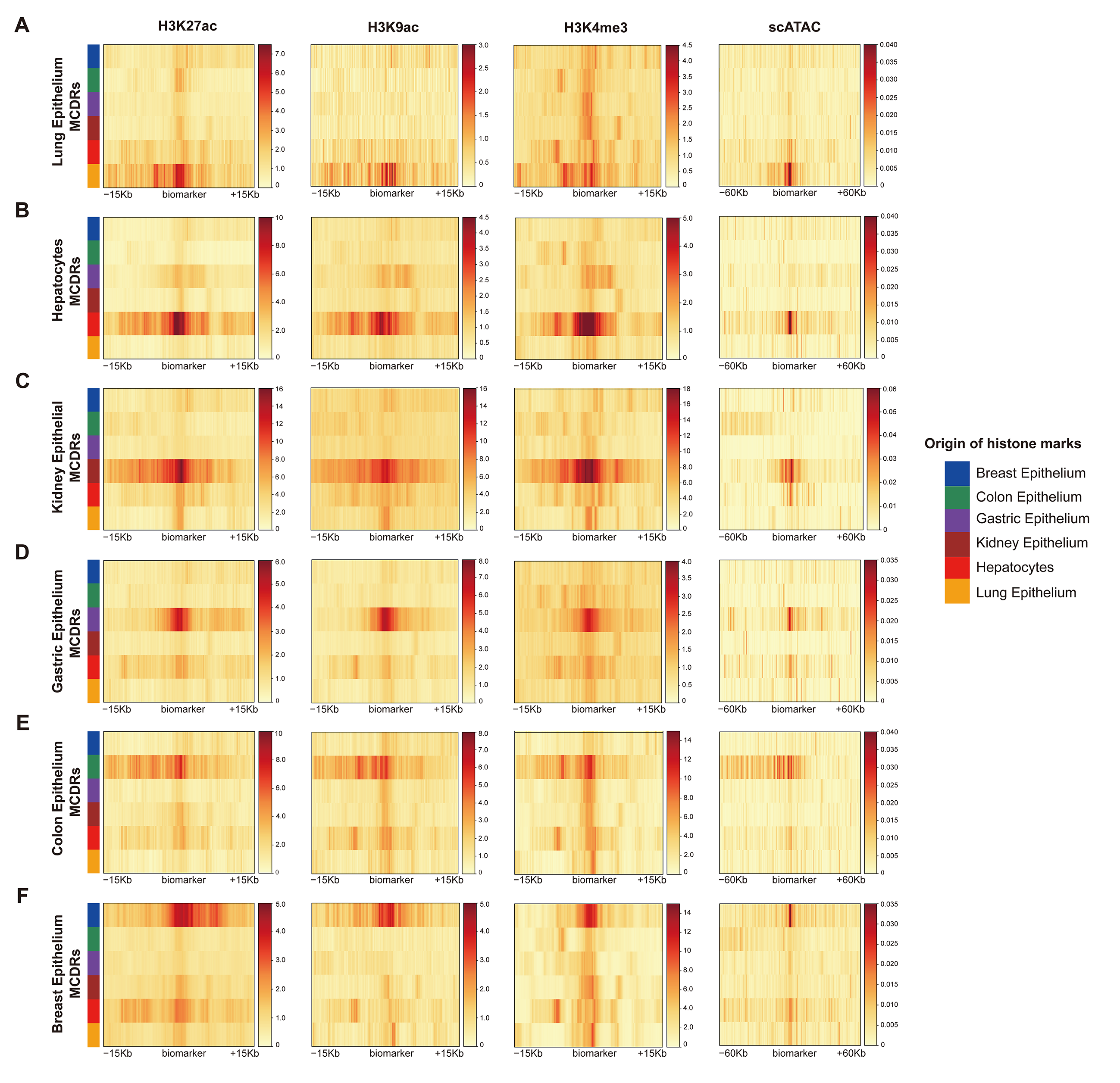

Supplement: Supplementary file 1 [file ijms-26-07199-s001.zip › Supplementary Figure S1.tif]

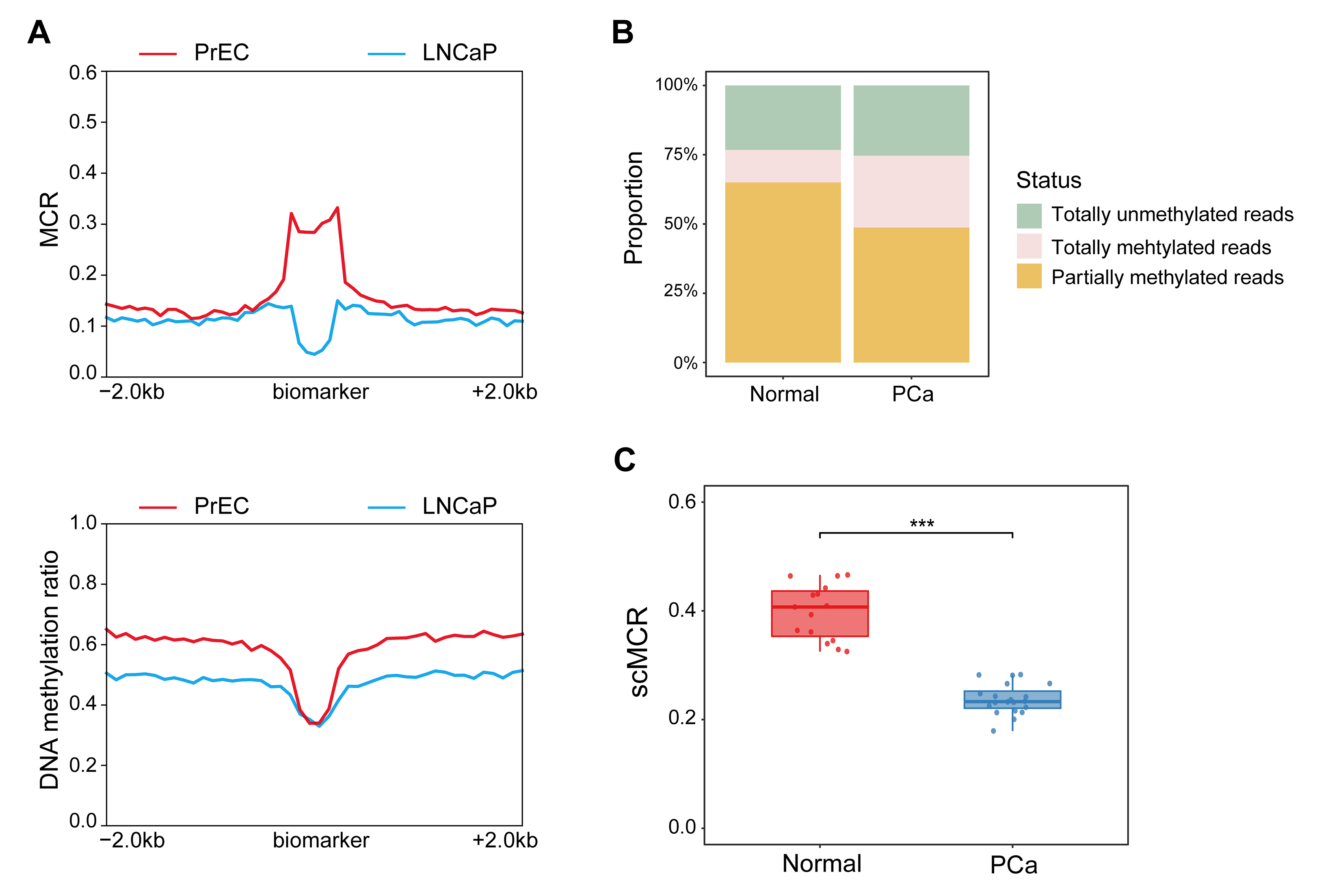

Supplement: Supplementary file 1 [file ijms-26-07199-s001.zip › Supplementary Figure S10.tif]

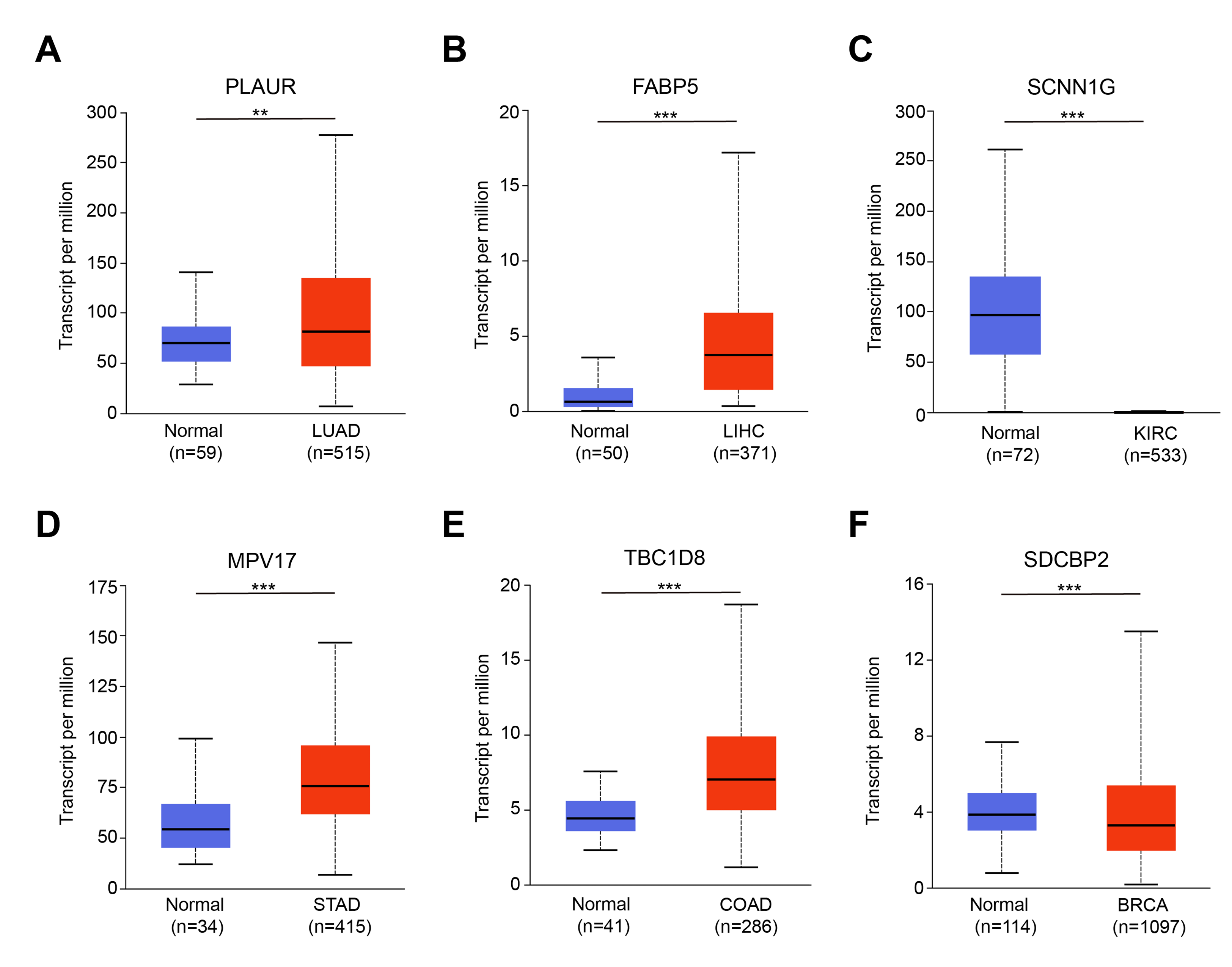

Supplement: Supplementary file 1 [file ijms-26-07199-s001.zip › Supplementary Figure S11.tif]

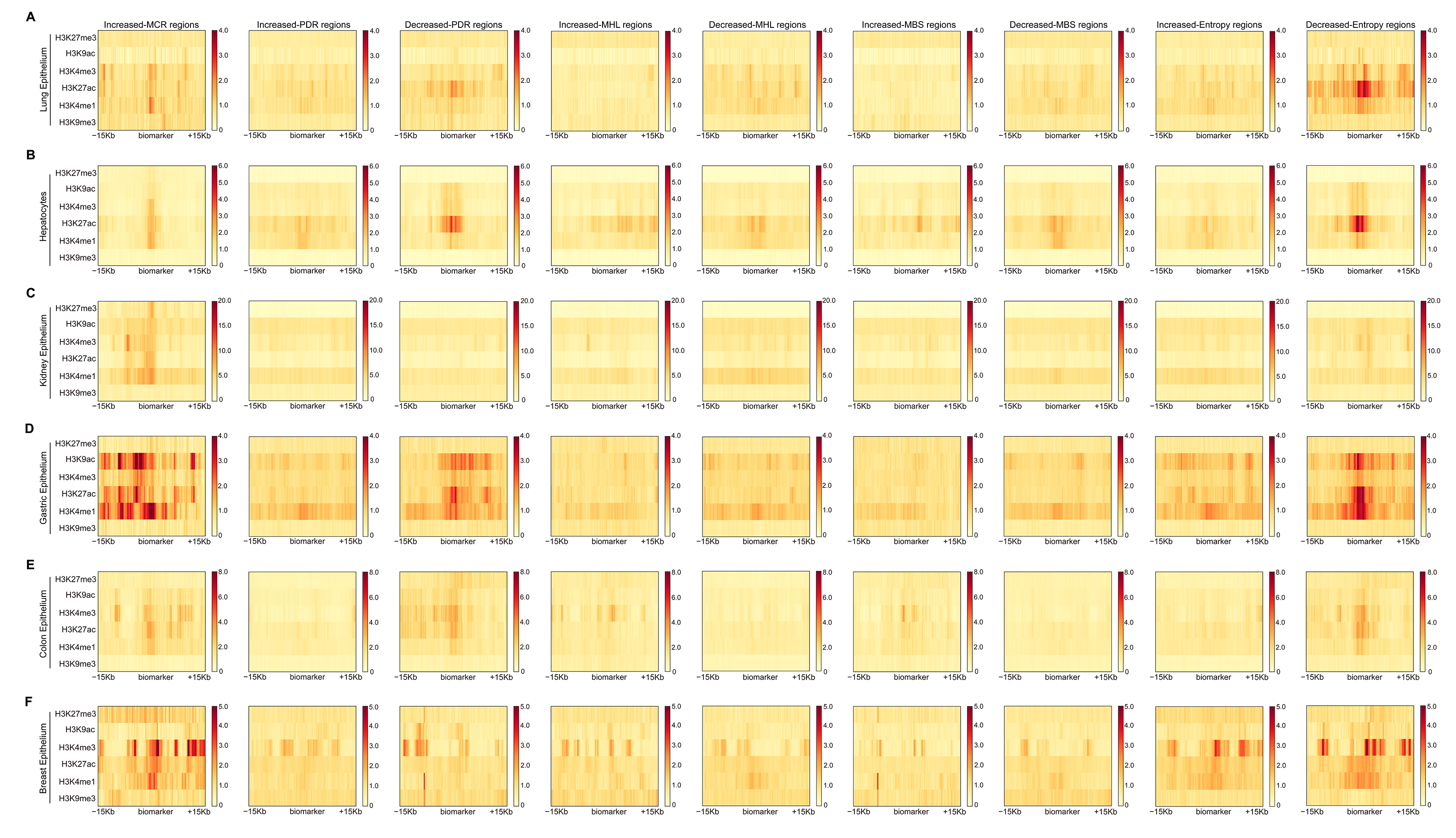

Supplement: Supplementary file 1 [file ijms-26-07199-s001.zip › Supplementary Figure S2.tif]

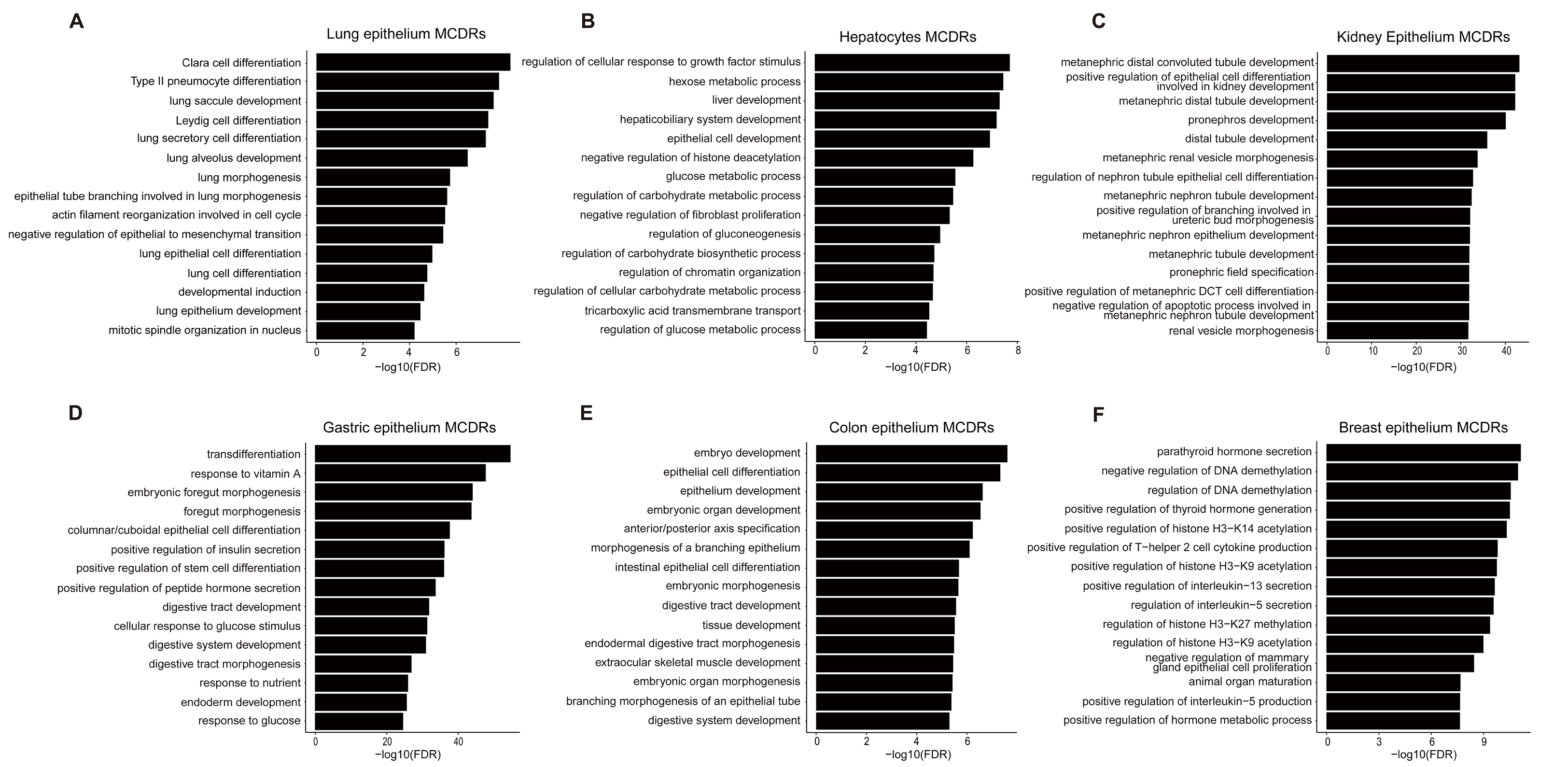

Supplement: Supplementary file 1 [file ijms-26-07199-s001.zip › Supplementary Figure S3.tif]

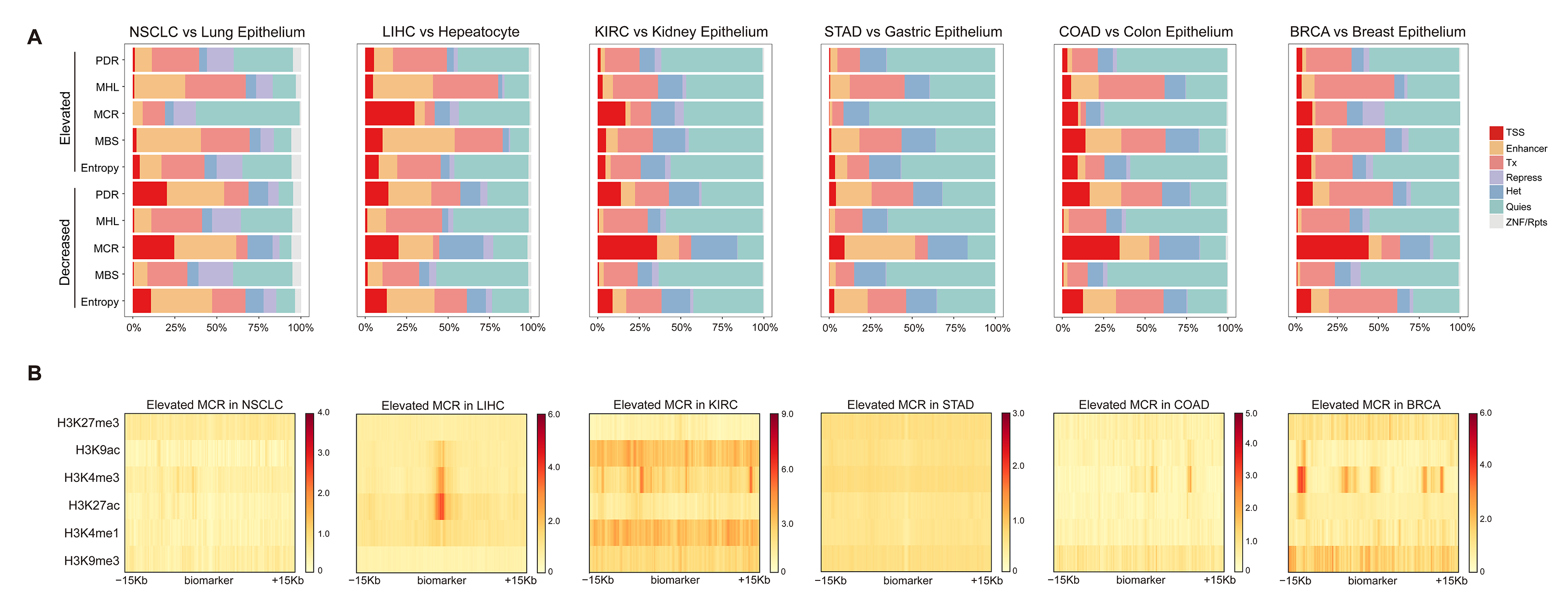

Supplement: Supplementary file 1 [file ijms-26-07199-s001.zip › Supplementary Figure S4.tif]

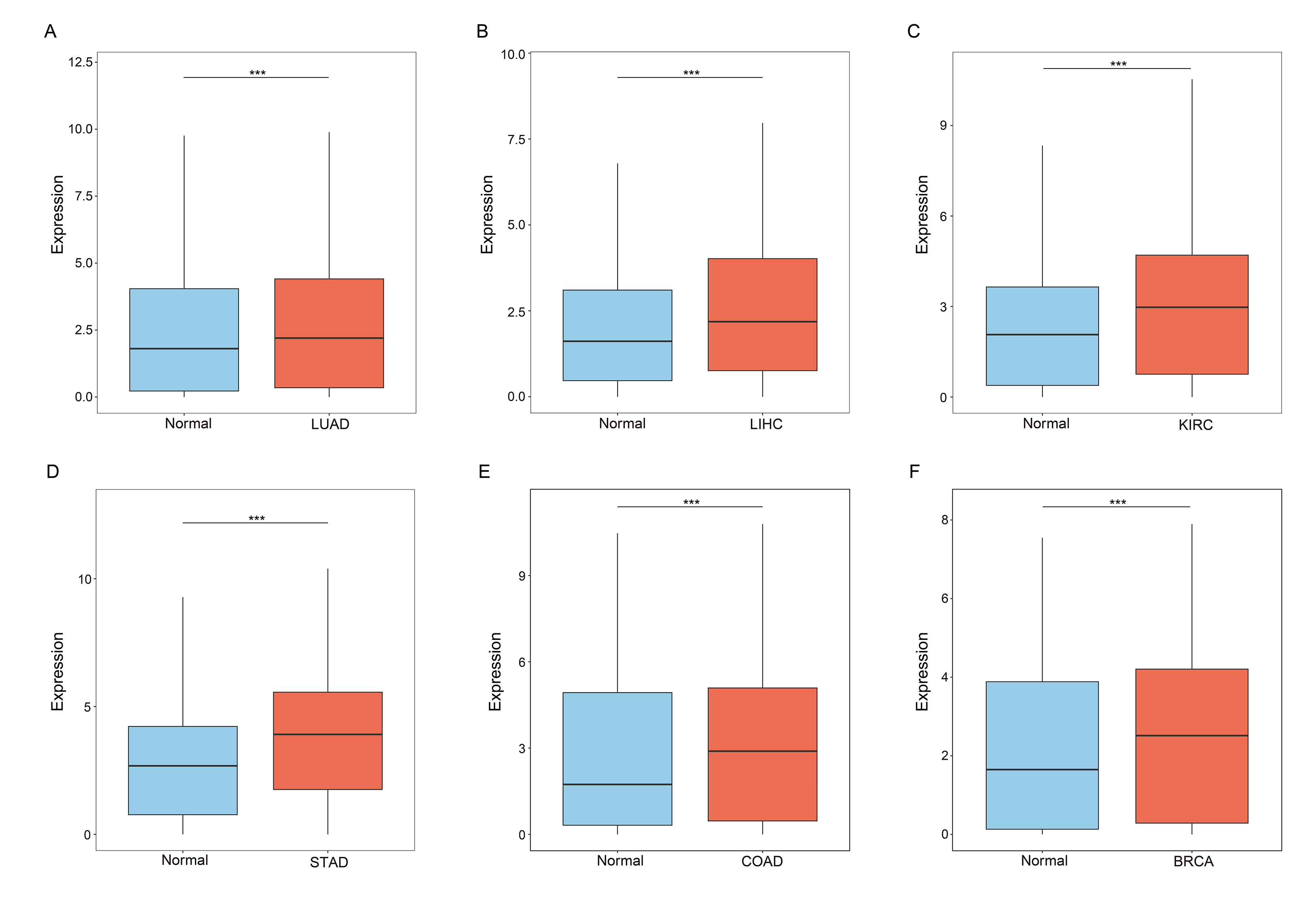

Supplement: Supplementary file 1 [file ijms-26-07199-s001.zip › Supplementary Figure S5.tif]

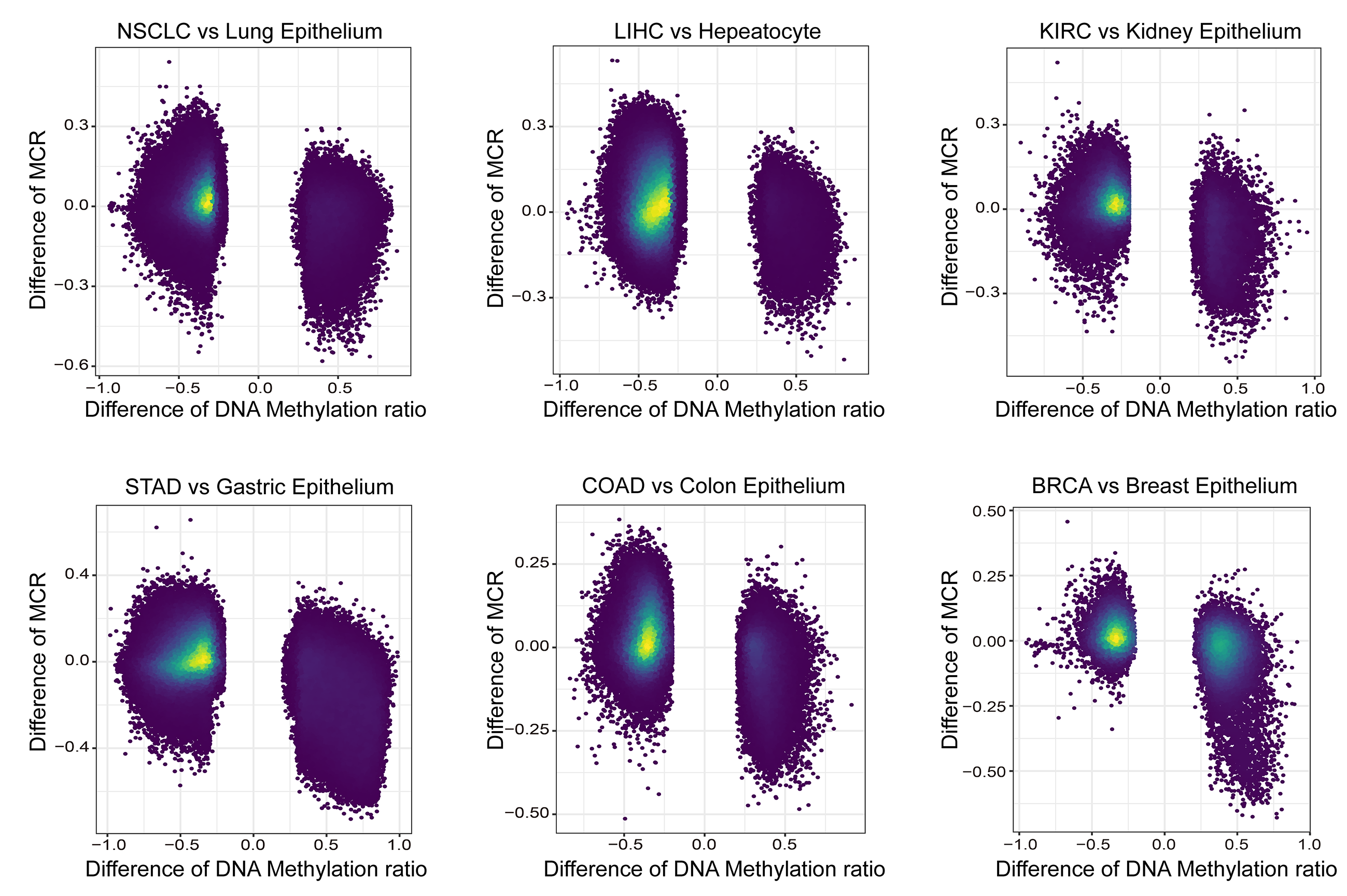

Supplement: Supplementary file 1 [file ijms-26-07199-s001.zip › Supplementary Figure S6.tif]

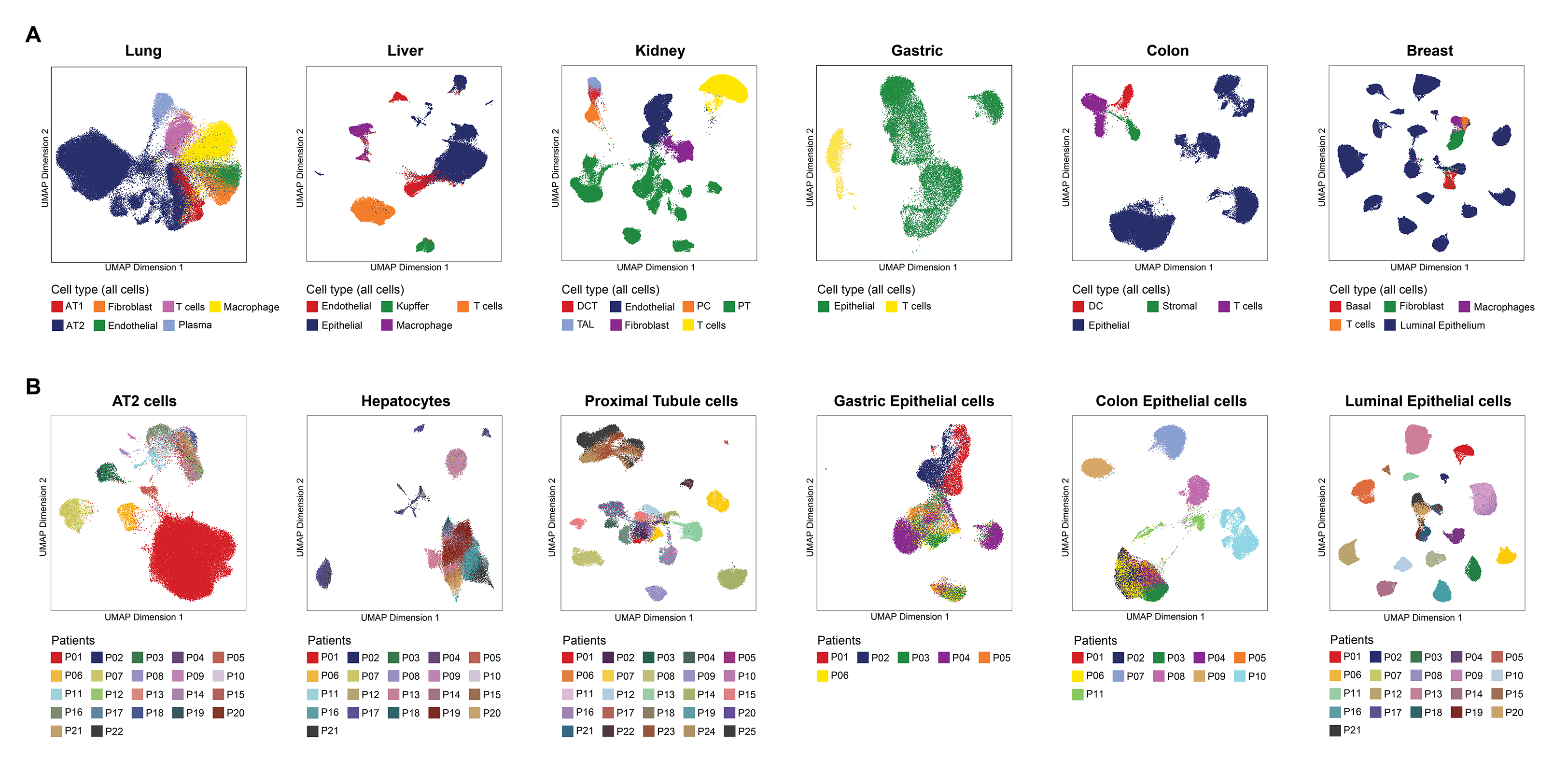

Supplement: Supplementary file 1 [file ijms-26-07199-s001.zip › Supplementary Figure S7.tif]

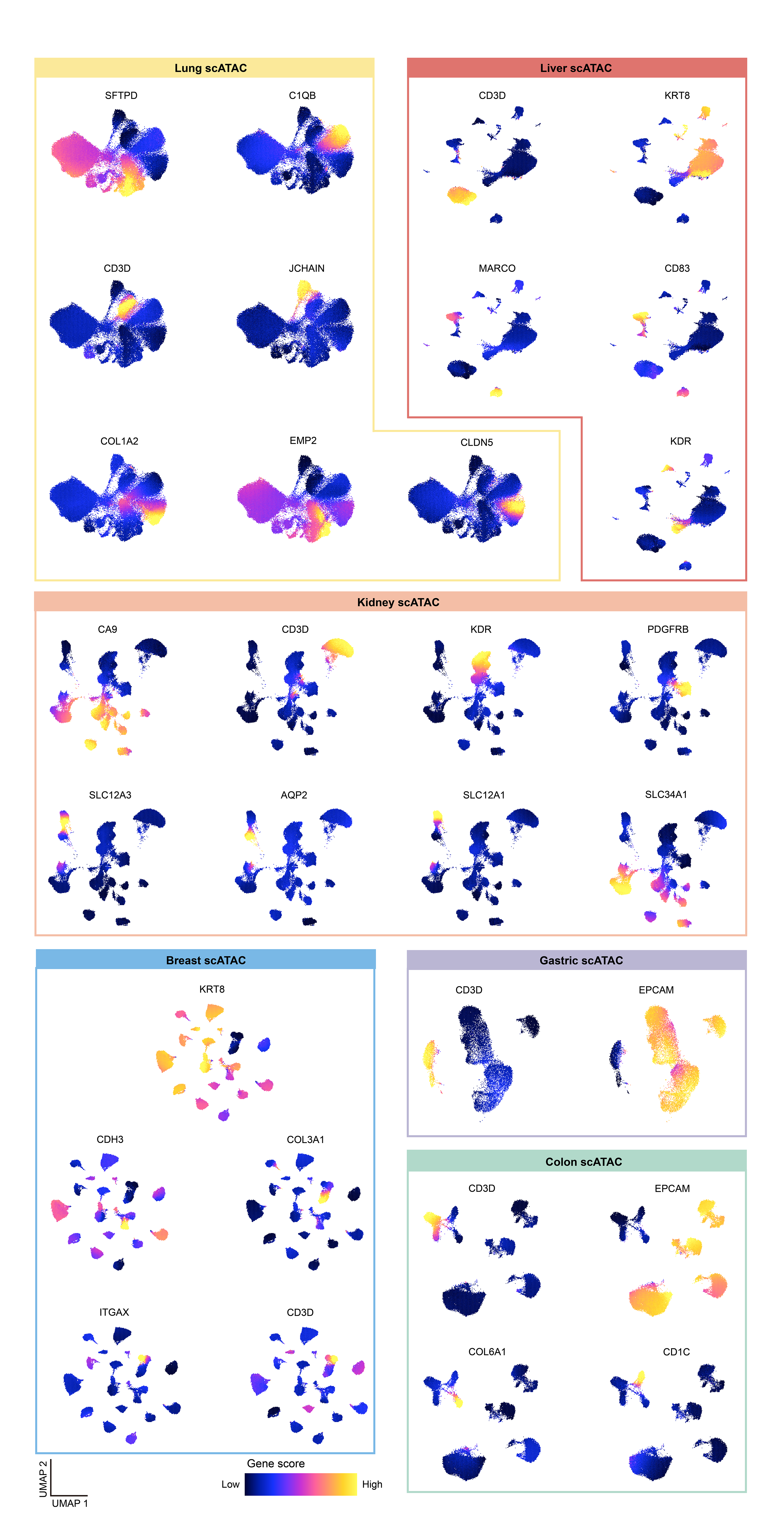

Supplement: Supplementary file 1 [file ijms-26-07199-s001.zip › Supplementary Figure S8.tif]

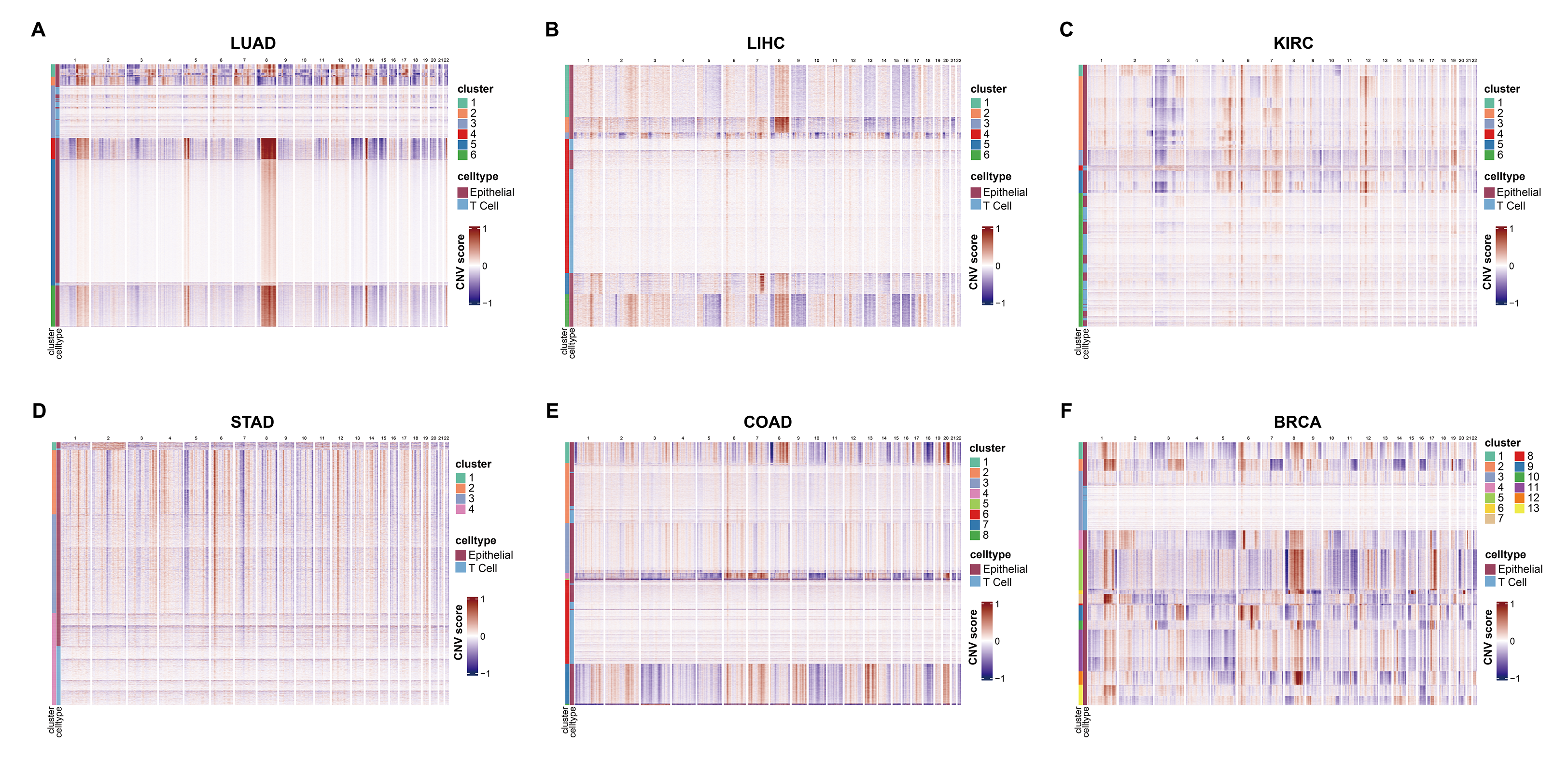

Supplement: Supplementary file 1 [file ijms-26-07199-s001.zip › Supplementary Figure S9.tif]
